# Anti-*Pseudomonas aeruginosa* activity of natural antimicrobial peptides when used alone or in combination with antibiotics

**DOI:** 10.3389/fmicb.2023.1239540

**Published:** 2023-09-05

**Authors:** Xueqi Chen, Shan Su, Yan Yan, Limei Yin, Lihong Liu

**Affiliations:** ^1^Department of Pharmacy, China-Japan Friendship Hospital, Beijing, China; ^2^Department of Pharmacy, The Affiliated Taian City Central Hospital of Qingdao University, Taian, China

**Keywords:** antimicrobial peptides, *Pseudomonas aeruginosa*, *Escherichia coli*, drug resistance, biofilms, interaction

## Abstract

The World Health Organization has recently published a list of 12 drug-resistant bacteria that posed a significant threat to human health, and *Pseudomonas aeruginosa* (*P. aeruginosa*) was among them. In China, *P. aeruginosa* is a common pathogen in hospital acquired pneumonia, accounting for 16.9–22.0%. It is a ubiquitous opportunistic pathogen that can infect individuals with weakened immune systems, leading to hospital-acquired acute and systemic infections. The excessive use of antibiotics has led to the development of various mechanisms in *P. aeruginosa* to resist conventional drugs. Thus, there is an emergence of multidrug-resistant strains, posing a major challenge to conventional antibiotics and therapeutic approaches. Antimicrobial peptides are an integral component of host defense and have been found in many living organisms. Most antimicrobial peptides are characterized by negligible host toxicity and low resistance rates, making them become promising for use as antimicrobial products. This review particularly focuses on summarizing the inhibitory activity of natural antimicrobial peptides against *P. aeruginosa* planktonic cells and biofilms, as well as the drug interactions when these peptides used in combination with conventional antibiotics. Moreover, the underlying mechanism of these antimicrobial peptides against *P. aeruginosa* strains was mainly related to destroy the membrane structure through interacting with LPS or increasing ROS levels, or targeting cellular components, leaded to cell lysis. Hopefully, this analysis will provide valuable experimental data on developing novel compounds to combat *P. aeruginosa*.

## 1. Background

As a common opportunistic gram-negative pathogen, *Pseudomonas aeruginosa* (*P. aeruginosa*) is a significant cause of nosocomial infection, leading to a variety of infections such as pneumonia, bacteremia, and urinary tract infections (Terzi et al., 2014; Yaeger et al., 2021). Individuals with weakened immune systems, such as those with metabolic diseases, hematological diseases, and malignant tumors, are more susceptible to *P. aeruginosa* infection (Burrows, 2018; Yaeger et al., 2021). The drugs commonly used for treating *P. aeruginosa* infection in clinical settings include penicillin, cephalosporins, aztreonam, aminoglycosides, fluoroquinolones and carbapenems (Moore and Flaws, 2011; Jangra et al., 2022). However, due to the inappropriate use of antibiotics, the resistance of *P. aeruginosa* to carbapenems and many other antibacterial drugs has increased rapidly, making the number of drug-resistant strains continue to arise and bringing challenges to clinical treatment (Hancock and Speert, 2000; Jangra et al., 2022). Therefore, there is an urgent need to develop new strategies to combat resistant *P. aeruginosa* infections.

The natural immune system serves as a functional and physiological barrier against microbial infections. Within this system, there are important effectors called antimicrobial peptides (AMPs) or host defense peptides (HDPs). Studies have shown that AMPs not only play a role in the regulation of inflammatory response, immune system, and apoptosis pathway, but they also exhibit a broad spectrum of antimicrobial activities (Su et al., 2010; Cho et al., 2012; Hancock et al., 2012; Buda De Cesare et al., 2020). Currently, more than 3,000 natural AMPs derived from microorganisms, plants, mammals, amphibians, and insects have been registered in the Antimicrobial Peptide Database (unmc.edu) (Bin Hafeez et al., 2021).

Most AMPs are composed of 10–100 amino acids and have an amphiphilic conformation, which can bind to the negatively charged components of bacterial cell surface, and integrate with the lipid bilayer, or enter the cytoplasm through the cell membrane, thus exhibit great antimicrobial activity against pathogens (Huan et al., 2020; Lachowicz et al., 2020). Multiple studies have demonstrated that many AMPs possess good antibacterial properties on their own. For example, BCp12 had been found to possess obvious antimicrobial activity against *Staphylococcus aureus*, *Listeria monocytogenes*, *Escherichia coli* (*E. coli*) and *Salmonella typhimurium* strains, with minimum inhibitory concentrations (MICs) of 0.4–1.6 mg/mL. Moreover, BCp12 induced low hemolytic activity and cytotoxicity on mammalian cells (Zhao et al., 2020). Furthermore, the combination of AMPs and traditional antibiotics demonstrated synergistic antimicrobial effects, and the combination not only enhanced the effectiveness of the two, but also expanded the antimicrobial spectrum of antibiotics. Notably, L_1_G, L_7_A and L_1_GA_5_K possessed good bioactivity, mild cytotoxicity, and high stability, and could rapidly kill bacteria by membrane rupture and intracellular materials release, with a MIC of 4–32 μM. Moreover, when combined with rifampicin, polymyxin B, and gentamicin, they exhibited either synergistic or additive effects against gram-negative bacteria (Zhu et al., 2021). AMPs may exert their antibacterial effects through different mechanisms, either by directly interacting with the bacterial membrane or by targeting other cellular components. The membrane-mediated mechanisms of action can be described by models: (1) the toroidal pore model, AMPs can enter the membrane vertically, the lipids would be dragged and bended by AMPs when they form the toroidal pore; (2) the barrel-stave model, AMPs can insert into the membrane, forming barrels and opening stable and transmembrane pores, thereby destabilizing the membrane potential and promoting leakage of ions and biomolecules; (3) the carpet-stave model, AMPs can act as detergents by localizing on the horizontal plane of the plasma membrane, then causing alterations and destruction; (4) the aggregate channel model, AMPs can competitively displace lipopolysaccharides (LPS)-associated divalent cations, leading to nonstructural aggregation of AMPs and lipids that disrupts the outer and inner membranes (Lee et al., 2011; Huan et al., 2020; Roque-Borda et al., 2021). In the non-membrane-targeted mechanism of action of AMPs, they can enter cells by direct penetration or endocytosis, and achieve antimicrobial activity through several mechanisms: (1) affecting nucleic acid and protein synthesis; (2) inhibiting enzyme activity and energy metabolism; (3) destroying cellular organelles; (4) producing oxidative stress response (Nguyen et al., 2011; Lei et al., 2019; Saeed et al., 2022).

In this review, we have presented a comprehensive summary of the inhibitory effects of natural AMPs used alone, and in combination with other drugs against *P. aeruginosa* planktonic cells and biofilms in Tables 1, 2. Moreover, we also summarized the relevant antibacterial mechanisms of AMPs, although they still needed to be further investigated. We believe this review will provide valuable experimental data for developing novel anti-*P. aeruginosa* agents.

**Table 1:** Antimicrobial peptides (AMPs) with activity against *Pseudomonas aeruginosa*.

Classification	Source	Code (ID)	AMPs	Molecular weight (kDa)	Strains	Inhibitory activities	Proposed Mechanism of Action	References
*Microorganisms*	*Bacteria*	AP02929	Lynronne-1	2.83	*P. aeruginosa* (n = 10)	MIC = 16–64 μg/mL	Reduces *arcA*, *arcB* and *arcC* expression	Oyama et al. (2017)
	*Bacteria*	AP02930	Lynronne-2	2.86		MIC = 32–256 μg/mL		
	*Bacteria*	AP02931	Lynronne-3	3.08		MIC = 64–512 μg/mL		
	*Bacteria*	AP02939	P15s	2.86	*P. aeruginosa* (n = 8)	MIC = 64–512 μg/mL	Reduces *arcD* expression	Mulkern et al. (2022)
	*Lactobacillus acidophilus* ATCC 4356	–	Acidocin 4,356	8.5	*P. aeruginosa* ATCC 27853	MIC_90_ = 128.22 μg/mL ACD (128.22 μg/mL) → killed >90% biofilm; ACD (256.44 μg/mL) → eradicated large parts of biofilm	Enhances bacterial membrane perturbation, inhibits virulence factors	Modiri et al. (2020)
	*Actinomyces ruminicola*	AP03310	Actifensin	4.1	*P. aeruginosa ATCC* 27853	MIC/MBC = 1,448 μg/mL Actifensin (724 μg/mL) → reduced >70% biofilm	Increases ROS production, disrupts cell membrane	Gbala et al. (2022)
*Plants*	*Spinach*	–	Defensin-d2	5.8	*P. aeruginosa* ATCC 27853	MIC = 7.5 μg/mL MBC = 123 μg/mL Defensin-d2 (3.75 μg/mL) → reduced >70% biofilm	Increases ROS production, disrupts cell membrane	Gbala et al. (2022)
	*Medicago Truncatula*	AP02428	Core MtDef4	1.98	*P. aeruginosa*	IC_50_ = 1.7–4.2 μM	Damages bacterial outer membranes	Sathoff et al. (2020), Velivelli et al. (2018)
	*Medicago Truncatula*	–	Core MtDef5	2.04	*P. aeruginosa*	IC_50_ = 8.5–14.6 μM	Interferes with DNA synthesis and transcription	
*Mammals*	*Porcine*	AP00195	Porcine protegrin-1	2.59	*P. aeruginosa* (n = 5)	MIC = 0.3–0.8 μg/mL	Disrupts cell membrane	Turner et al. (1998)
	*Human*	–	FLG2-4	70–140	*P. aeruginosa* ATCC33354	MEC = 0.4 μM	Induces membrane blebbing, impairs DNA polymerases processivity, thereby stops bacterial replication	Hansmann et al. (2015)
	*Human*	–	LL-37	4.493	*P. aeruginosa* (*n* = 6)	MIC = 8–32 μg/mL LL-37 (16 μg/mL) → inhibited 80% biofilm formation; LL-37 (4 μg/mL) → made pre-formed biofilm thinner	Reduces bacterial cells attachment, stimulates motility twitch, suppresses the expression of key QS signaling molecules, downregulates biofilm formation-related genes expression	Turner et al. (1998), Overhage et al. (2008)
	*Cattle*	AP00366	BMAP-27	3.47	*P. aeruginosa* strains (*n* = 25)	MIC_90_/ MBC_90_ = 16 μg/mL BMAP-27 (8 μg/mL) → reduced biofilm formation; BMAP-27 (80 μg/mL) → against pre-formed biofilms	Induces of membrane permeabilization	Skerlavaj et al. (1996), Pompilio et al. (2012)
	*Cattle*	AP00367	BMAP-28	3.32		MIC_90_/MBC_90_ = 32 μg/mL BMAP-28 (16 μg/mL) → reduced biofilm formation; BMAP-28 (160 μg/mL) → against pre-formed biofilms	Induces of membrane permeabilization	
	*Human*	–	Human β-defensin 2	4–5	*P. aeruginosa*	LD_90_ = 10 μg/mL HBD2 (0.25–0.5 μM) → reduced 75% biofilm	Induces the change of the biofilm surface topology	Schröder and Harder (1999), Parducho et al. (2020)
*Insects*	*Apis mellifera*	–	Jelleines I	0.95	*P. aeruginosa* ATCC 27853	MIC = 10 μg/mL	Disrupts cell membrane	Fontana et al. (2004)
	*Apis mellifera*	–	Jelleines II	1.05		MIC = 15 μg/mL	–	
	*Apis mellifera*	–	Jelleines III	1.08		MIC = 30 μg/mL	–	
	*Hermetia Illucens*	–	Hill-Cec 1	4.79	*P. aeruginosa* (*n* = 4)	MIC = 1 μM IC_50_ (1.3 ± 0.57 μM) → reduced 50% biofilm mass; IC_50_ (2.1 ± 0.52 μM) → reduced 50% biofilm visibility	Causes membrane depolarization and membrane damage	Van Moll et al. (2022)
	*Hermetia Illucens*	–	Hill-Cec 10	5.28		MIC = 1 μM IC_50_ (7.5 ± 3.5 μM) → reduced 50% biofilm mass; IC_50_ (11 ± 1.7 μM) → reduced 50% biofilm visibility	Causes membrane depolarization and membrane damage	
*Amphibians*	*Phyllomedusa hypochondrialis frogs*	–	Dermaseptin K_4_K_20_S_4_	2.88	*P. aeruginosa* PA01	MIC = 0.39 μg/mL	Breaks down membrane lipids and disperses bacteria	Zaïri et al. (2014)
	*Phyllomedusa hypochondrialis frogs*	–	Dermaseptin K_4_S_4_	2.86		MIC = 12.5 μg/mL	Breaks down membrane lipids and disperses bacteria	
	*Limnonectes kuhlii Frog*	AP02106	Temporin-LK1	1.95	*P. aeruginosa* (*n* = 2)	MIC = 2.5 μg/mL	–	Wang et al. (2013)
	*Limnonectes kuhlii Frog*	AP02110	Rugosin-LK2	3.46		MIC = 2.5 μg/mL	–	
	*Limnonectes kuhlii Frog*	AP02109	Rugosin-LK1	3.52		MIC = 5 μg/mL	–	
	*Limnonectes kuhlii Frog*	AP02107	Gaegurin-LK1	2.57		MIC = 5–10 μg/mL	–	
	*Limnonectes kuhlii Frog*	AP02108	Gaegurin-LK2	2.52		MIC = 5–10 μg/mL	–	
	*Green edible frog Pelophylax lessonae*	–	Esculentin (1–21)	2.61	*P. aeruginosa* (*n* = 9)	MIC = 4 μM MBEC = 6 μM MBCb = 12 μM	Penetrates biofilm plasma membrane, and causes β-galactosidase release	Luca et al. (2013)
	*Hylarana latouchii*	–	Brevinin-1HL	2.5	*P. aeruginosa* ATCC27853	MIC = 256 μg/mL MBIC = 256 μg/mL	Destroys cell membranes and binds to bacterial DNA	Lin et al. (2021)
	*Hylarana latouchii*	–	Temporin-HLa	1.88		MIC >512 μg/mL	Destroys cell membranes and binds to bacterial DNA	
	*Hylarana latouchii*	–	Temporin-HLb	1.5		MIC >512 μg/mL	Destroys cell membranes and binds to bacterial DNA	
*Others*	*Fish*	–	Gaduscidin-1	2.49	*P. aeruginosa* PAO1	MIC = 2 μM Gad-1 (64 μM) → cleared 60–72% biofilm; Gad-1 (32–64 μM) → inhibited biofilm formation completely	Reduces *P. aeruginosa* viability in biofilms, cleaves eDNA	Portelinha and Angeles-Boza (2021)
	*Morone saxatilis*	AP00473	Piscidin 1	3	*P. aeruginosa* PA01	MIC = 16 μM	Disrupts cell membranes	Salger et al. (2016)
	*Morone saxatilis*	AP00474	Piscidin 3	2.92		MIC = 32 μM	Possesses nuclease activity and destroys eDNA	
	*Alligator mississippiensis*	DRAMP20805	Apo5	3.13	*P. aeruginosa* (*n* = 2)	EC_50_ = 0.0878–0.467 μg/mL	Disrupts cell membranes	Barksdale et al. (2016)
	*Alligator mississippiensis*	DRAMP20806	Apo6	2.79		EC_50_ = 0.13–1.17 μg/mL	Disrupts cell membranes	
	*Alligator mississippiensis*	AP02517	A1P	4.13	*P. aeruginosa* PAO1	EC_50_ = 38.6 μg/mL	–	
	*Alligator mississippiensis*	–	APOC1_64–88_ APOC1_67-88_ A1P_394–428_ FGG_398–413_ FGG_401-413_	3.1 2.77 4.11 3.93 1.56	*P. aeruginosa*	EC_50_ = 0.948–11.1 μM	–	Bishop et al. (2015)
	*Silkworm Bombyx mori*	AP01259	ABP-CM4	3.79	*P. aeruginosa* ATCC 27853	MIC = 16 μM	Destroys cell membrane and interacts with DNA	Li et al. (2020)
	*Brown* *garden snail*	–	*Helix aspersa* mucus	30–100	*P. aeruginosa* (*n* = 2)	Measurable zones of inhibition (11.12 mm and 11.63 mm) were observed	–	Pitt et al. (2015)
	*Brown* *garden snail*	–	*Cornu aspersum* mucus	17.5–37.4	*P. aeruginosa* (*n* = 7)	Mean zones of inhibition recorded were all between 9 and 13 mm	–	Pitt et al. (2019)

MIC, minimum inhibitory concentration; MBC, minimum bactericidal concentration; MEC, minimal effective concentration; EC_50_, half-maximal effective concentrations; IC_50_, half maximal inhibitory concentration; LD_90_: 90% lethal dose; MBEC, minimum concentration preventing re-growth of bacteria from the treated biofilm, within 4 h; MBCb, minimum concentration required to reduce the number of viable biofilm cells of ≥ 3 log10 (99.9% killing) after 2 h; MBIC, minimum biofilm inhibitory concentration; Ref, reference; −, unknown.

**Table 2:** The anti-*Pseudomonas aeruginosa* activity of antimicrobial peptides (AMPs) when used in combination with drugs.

Source	AMPs	Code (ID)	Molecular weight (kDa)	Agents	Strains	Cell types	Antimicrobial effects		References
							FICI	Interpretation	
*Microorganism*	Nisin	–	3.35	Colistin	*P. aeruginosa* (*n* = 6)	Planktonic cells	0.375–0.625	SYN (4), ADD (2)	Jahangiri et al. (2021)
*Human*	P10	–	3.11	Ceftazidime	*P. aeruginosa* (n = 6)	Planktonic cells	0.375–0.75	SYN (4), ADD (2)	Jahangiri et al. (2021)
				Doripenem			0.5–0.625	SYN (5), ADD (1)	
	LL-37	–	4.49	Colistin	*P. aeruginosa* (n = 2)	Planktonic cells	0.38- > 0.5	SYN (1), ADD (1)	Han et al. (2022)
				Vancomycin			≥0.5	SYN (1), ADD (1)	
				Polymyxin B			0.38- > 0.5	SYN (1), ADD (1)	
				Ciprofloxacin	*P. aeruginosa* (n = 4)	Biofilms	–	SYN (4), ADD (1)	Dosler and Karaaslan (2014)
				Tobramycin			–	SYN (2), ADD (3)	
				Colistin			-	SYN (2), ADD (3)	
*Frog*	Magainin II	AP00144	2.48	Rifampicin	*P. aeruginosa* (*n* = 2)	Planktonic cells	0.312	SYN	Cirioni et al. (2008)
	Lys-[Trp6] hy-a1	–	1.87	Ciprofloxacin	*P. aeruginosa* ATCC 9027	Planktonic cells	0.37	SYN	Carneiro et al. (2020)
						Biofilms	0.5625	ADD	
	Ocellatins-PT3	–	2.53	Ciprofloxacin	*P. aeruginosa* (*n* = 2)	Planktonic cells	0.25–0.38	SYN	Bessa et al. (2018)
				Ceftazidime			0.38–0.5	SYN	
	Citropin 1.1	AP00351	1.62	Colistin	*P. aeruginosa* (*n* = 3)	Planktonic cells	0.26–0.75	SYN (2), ADD (1)	Jorge et al. (2017)
						Biofilms	–	SYN (2), IDD (1)	
	Temporin A	AP00094	1.4			Planktonic cells	0.27–0.75	SYN (2), ADD (1)	
						Biofilms	–	SYN (2), ADD (1)	
*Cationic AMP*	Colistin	AP02204	1.22	NCL195	*P. aeruginosa* (*n* = 25)	Planktonic cells	0.12–0.5	SYN	Nguyen et al. (2021)
				Ciprofloxacin	*P. aeruginosa*	Biofilms	–	SYN	Pamp et al. (2008)
				Tetracycline		Biofilms	–	SYN	
*Crab*	Tachyplesin I	AP00214	2.27	Colistin	*P. aeruginosa* (*n* = 3)	Planktonic cells	0.38–0.63	SYN (2), ADD (1)	Jorge et al. (2017)
						Biofilms	–	SYN (1), ADD (2)	
	Sphistin	AP02814	3.96	Azithromycin	*P. aeruginosa* ATCC 9027	Planktonic cells	0.35	SYN	Liu et al. (2020)
				Rifampicin			0.3125	SYN	
*Bee*	Melittin	AP00146	2.87	Doripenem	*P. aeruginosa* (*n* = 5)	Planktonic cells	0.01–0.06	SYN	Akbari et al. (2019)
				Ceftazidime			0.02–0.5	SYN	
*Bovine*	LfcinB (20–25)_4_	–	4.84	Ciprofloxacin	*P. aeruginosa* ATCC 27853	Planktonic cells	0.09	SYN	Vargas-Casanova et al. (2019)
*King Cobra*	OH-CATH30	AP00923	3.61	Ciprofloxacin	*P. aeruginosa* (*n* = 4)	Planktonic cells	0.375–0.5	SYN	Li et al. (2014)
				Levofloxacin			0.375–0.5	SYN	
*Aedes aegypti*	Cecropin A2	–	3.6	Tetracycline	*P. aeruginosa* (*n* = 18)	Planktonic cells	0.25	SYN	Zheng et al. (2017)
*Giant silk moth*	Cecropin A	AP03235	3.88	Rifampicin	*P. aeruginosa* (*n* = 2)	Planktonic cells	0.312	SYN	Cirioni et al. (2008)
*Amide*	CAMA	–	1.77	Ciprofloxacin	*P. aeruginosa* (*n* = 4)	Biofilms	-	SYN	Dosler and Karaaslan (2014)
*Synthetic*	AMP38	–	1.25	Imipenem	*P. aeruginosa* (*n* = 4)	Planktonic cells	0.07–0.18	SYN	Rudilla et al. (2016)
						Biofilms	0.25	SYN	
*Synthetic*	OW peptide	–	0.99	Ampicillin	*P. aeruginosa* ATCC 2114	Planktonic cells	0.38	SYN	Al Tall et al. (2019)
				Chloramphenicol			0.41	SYN	
*Synthetic*	Lin-SB056-1	–	1.26	EDTA	*P. aeruginosa* (*n* = 2)	Biofilms	-	SYN	Maisetta et al. (2017)
*Synthetic*	P5	–	2.36	Meropenem	*P. aeruginosa* M13513	Planktonic cells	–	SYN	Martinez et al. (1861)
*Synthetic*	Melimine	AP02709	3.8	Ciprofloxacin	*P. aeruginosa* ATCC 27853	Biofilms	–	SYN	Yasir et al., 2020

FICI, fractional inhibitory concentration index; SYN, synergy; ADD: addition; Ref, reference; −, unknown.

## 2. Anti-*Pseudomonas aeruginosa* activity of AMPs

The AMP database has documented many AMPs that have been derived from various lives, including 335 bacteriocins from bacteria, 4 AMPs from archaea, 8 AMPs from protozoa, 13 AMPs from fungi, 342 AMPs from plants, 2,200 AMPs from animals, and some synthetic peptides (Zhu et al., 2017; Boparai and Sharma, 2020a; Perez-Rodriguez et al., 2022a). In this review, our focus is on AMPs that have anti-*P. aeruginosa* effects, and we have categorized them into six different groups according to their origins: *microorganisms*, *plants*, *mammals*, *insects*, *amphibians*, and a varied group of *others*. In Table 1, we summarized the inhibitory activity of natural AMPs against *P. aeruginosa* according to different sources, and ranked the antibacterial activity from strongest to weakest.

### 2.1. AMPs from *microorganisms*

Numerous researchers have found and isolated a variety of AMPs from *bacteria* and *fungi*, which have significant antibacterial activity against different types of pathogens (Andrä et al., 2001; Szekeres et al., 2005; Buda De Cesare et al., 2020; Boparai and Sharma, 2020b).

The rumen is a complex microbiome consisting of various microorganisms, including fungi, bacteria, and viruses (Morgavi et al., 2013; McCann et al., 2014; Oyama et al., 2017). Many researchers have regarded it as a potential resource for discovering new AMPs. For example, Oyama et al. and Mulkern et al. reported that Lynronne-1, 2, 3, and P15s (with 2.83–3.08 kDa in size) were identified from *bovine rumen microbiome* all possessed good antibacterial activity (Privé et al., 2015; Oyama et al., 2017; Mulkern et al., 2022). In these two studies, researchers found that the four peptides possessed significant *in vitro* activity against *P. aeruginosa* and *E. coli*, with MICs of 4–512 and 32–64 μg/mL, respectively. *In vivo* experiments, local administration of Lynronne-1 (10% w/v) significantly reduced the number of bacteria infected by methicillin-resistant *Staphylococcus aureus* (MRSA) in mice (Oyama et al., 2017). In addition, in *Galleria mellonella* (*G. mellonella*) infection model, treatment with Lynronne 1 and Lynronne 2 at 32 and 128 mg/kg resulted in a 100% survival rate of the larvae (Mulkern et al., 2022). Among them, Lynronne-2 had the highest safety, as it did not exhibit any cytotoxicity toward HUVEC and HepG2 cells at a concentration of 128 μg/mL. However, Lynronne-1 and Lynronne-3 showed some degree of toxicity, with their 50% lethal concentration (LC_50_) values of 98.1 and 128 μg/mL (Oyama et al., 2017). In addition to their ability to bind to bacterial membrane lipids and enhance membrane permeability, Lynronne-1 and 2 downregulated genes expression of *arcA*, *arcB*, and *arcC*, while P15s decreased *arcD* expression and indirectly affected arginine metabolism.

In addition to *rumen microbiome*, *bacteriocins* produced by *lactic acid bacteria* (LAB) had been extensively studied due to their safety and utility. Acidocin 4356 (ACD) is a novel bacteriocin with a size of 8.3 kDa, which was isolated from *Lactobacillus acidophilus ATCC 4356* (Modiri et al., 2020). ACD had antimicrobial activity against both *E. coli* and *P. aeruginosa*, causing 35 and 80% inhibition of their growth at the same concentration. As shown in Table 1, ACD had inhibitory effects against *P. aeruginosa* planktonic cells, with an MIC_90_ of 128.22 μg/mL. Additionally, ACD showed potent anti-*P. aeruginosa* biofilm activity, killing over 90% of biofilms at a concentration of 128.22 μg/mL and significantly reducing the pre-formed biofilms at a concentration of 256.44 μg/mL. In *in vivo* experiments, ACD was found to effectively suppress infection caused by *P. aeruginosa* in mice. Compared with the control group, the peptide-treated mouse lung slices exhibited the decreased recruitment of macrophages and lung cells, as well as a reduction in epithelial hyperplasia and structural degeneration. Mechanism of action experiments revealed that ACD not only enhanced bacterial membrane perturbation, but also reduced the production of *P. aeruginosa* virulence factors, such as pyoverdine siderophore, pyocyanin toxin, secretory protease, and elastase enzyme (Modiri et al., 2020). Moreover, it had a low hemolytic activity of less than 20% on human erythrocytes at a concentration of 400 μg/mL, indicating its potential as a promising therapeutic compound. Actifensin, a *bacteriocin* produced by *Actinomyces ruminicola* with a molecular weight of 4.1 kDa. It exhibited weak antibacterial activity against *P. aeruginosa* strains with MIC of 1448 μg/mL, whereas predominantly inhibited methicillin-resistant *S. aureus* and *Candida albicans* with MIC values of 23 μg/mL and 45 μg/mL. Its mechanism of anti-*P. aeruginosa* may be related to induce reactive oxygen species (ROS) generation, thereby destroyed cellular membrane permeability, but further molecular mechanisms were unclear (Sugrue et al., 2020; Gbala et al., 2022). More importantly, it has been found to have a high biosafety, and it causes less than 1.5% hemolysis on mouse erythrocytes in all tested concentrations.

### 2.2. AMPs from plants

*Plant* defensins are small cationic peptides with 5–7 kDa in size that contain multiple cysteine groups to safeguard plants against microbial invasion (Gbala et al., 2022). These peptides have a wide-ranging ability to inhibit the growth of various pathogens including filamentous fungi and bacteria (Perez-Rodriguez et al., 2022b), we summarized the data on the inhibitory activity of several *plant*-derived AMPs against *P. aeruginosa* in Table 1.

Many peptides found in *spinach leaves* exhibited antimicrobial activities. One such peptide is So-D1-7, which was effective to against gram-positive and gram-negative bacterial pathogens as well as fungi at concentrations below 20 μM (Segura et al., 1998). Another antibacterial component identified in *spinach leaves* was Defensin-d2, which had a molecular weight of 5.8 kDa. It had been found to be more active against *P. aeruginosa* than *E. coli in vitro*, with the MIC values of 7.5 and 30 μg/mL, respectively (Gbala et al., 2022). Defensin-d2 also had the ability to inhibit biofilm formation of *P. aeruginosa* in a concentration dependent manner. When treated with 3.75 μg/mL of defensin-d2 for 24 h, the mass percentage of biofilm was reduced by more than 70% (Gbala et al., 2022). The related mechanism was similar to that of actifensin, involving increased production of ROS production and disruption of cell membrane. The highest hemolysis rate of 2.89% was observed at the concentration of 985 μg/mL.

*Medicago truncatula*, a type of plant, contained various peptides that exhibited antimicrobial activities against human pathogens. These peptides interacted with the phospholipids of fungal cell membranes, causing an increase in membrane permeability and ultimately leading to fungi cell death (Sathoff et al., 2019). In a study conducted by Sathoff et al. it was found that core MtDef4 and MtDef5 (with the molecular weights of 1.98–2.04 kDa) derived from *Medicago truncatula*, were able to inhibit the growth of *P. aeruginosa* with IC_50_ = 1.7–4.2 and 8.5–14.6 μM, respectively (Sathoff et al., 2019, 2020. Moreover, Core MtDef4 could induce gene expression of the amino arabinose modification of LPS and surface polycation spermidine production operons, and it could damage outer membranes of *P. aeruginosa*, while MtDef5 appeared to interfere with DNA synthesis and transcription (Velivelli et al., 2018; Sathoff et al., 2020).

### 2.3. AMPs from mammals

AMPs derived from *human* play a crucial role in the human immune system, which can protect the body from pathogen invasion by reducing their virulence factors (Bosso et al., 2018). Therefore, the exploration of *human* peptides may be significant for developing antimicrobial drugs. As illustrated in Table 1, specific description of several AMPs derived from *mammals* with significant anti-*P. aeruginosa* activity are shown below.

Protegrins and their derivatives are a new class of AMP antibiotics derived from mammals, which have been proved to have a wide range of antimicrobial activities, including gram-positive and gram-negative bacteria as well as fungi (Bellm et al., 2000). Porcine protegrin-1 (PG-1), an AMP with a molecular weight of 2.59 kDa, had *in vitro* antimicrobial activities against gram-negative bacteria. It effectively inhibited the growth of *E. coli* and *P. aeruginosa* planktonic cells, with MICs of 0.2–0.5 and 0.3–0.8 μg/mL, respectively (Turner et al., 1998). The mechanistic studies revealed that PG-1 was capable of binding to the membrane, forming voltage-gated channels within the lipid bilayer and dissolving liposomes, ultimately leading to the disruption of the cell membrane and cell death (Bellm et al., 2000). The results of the cell cytotoxicity experiment showed that PG-1 had limited effects on mammalian cells, only reducing cell number at concentrations over 50 μg/mL (Morroni et al., 2019).

Apart from the physical skin barrier-stratum corneum, *human skin* also has an innate defense barrier. This barrier consists of AMPs and proteins, which help to control the growth of microorganisms on the body surface and reduce the risk of infection (Elias, 2005; Gallo and Hooper, 2012; Harder et al., 2013). Filaggrin-2 (FLG2), also known as ifapsoriasin, is expressed in *human skin* and serves to protect the skin from environmental damage (Wu et al., 2009). The radial diffusion assays provided evidence that FLG2-4, with a molecular mass ranging from 70 to 140 kDa, had powerful antimicrobial activities against *P. aeruginosa* and *E. coli* planktonic cells with MEC of 0.4 μM and 2.4 μM, respectively (Hansmann et al., 2015). Besides, the underlying anti-*P. aeruginosa* mechanism of FLG2-4 appears to involve inducing membrane blebbing, disrupting DNA polymerases activity, ultimately halting bacterial replication.

Cathelicidin LL-37 is a cationic AMP that is produced by *humans*, and has a molecular weight of 4.493 kDa. It is a broad-spectrum cathelicidin known for its strong chemotaxis and immunomodulatory properties. Besides, it plays a role in regulating the systems of immune, respiratory, gastrointestinal, and skin by participating in molecular pathways (Sørensen et al., 2001; Zanetti, 2005; Burton and Steel, 2009; Fabisiak et al., 2016).

LL-37 exhibited strong efficacy against most detected gram-negative bacteria. In broth microdilution assay, LL-37 demonstrated moderate antimicrobial activity against *E. coli* and *P. aeruginosa* with MIC values of 6–32 and 8–32 μg/mL, respectively (Turner et al., 1998). In addition, LL-37 displayed potent anti-biofilm activities, inhibiting 80% biofilm formation of *P. aeruginosa* at 16 μg/mL and reducing the thickness of pre-formed biofilm at 4 μg/mL (Overhage et al., 2008). The quorum sensing (QS) system was closely related to bacterial biofilms formation, the expression of virulence factors, and multiple drug resistance pathways (Al Akeel et al., 2019). There are two characteristic QS systems in *P. aeruginosa*: the Las system and the Rh1 system (Yu and Ma, 2017). LL-37 was found to inhibit *P. aeruginosa* biofilm formation through various mechanisms, including reducing bacterial cells attachment, stimulating motility twitch, suppressing the expression of key QS signaling molecules and significantly downregulating the expression of more than 50 biofilm formation-related genes, including *lasI* and *rhlR* genes (Overhage et al., 2008). However, it is important to note that LL-37 has high toxicity to mammalian cells. Therefore, further application of LL-37 may require structural modifications or combination with other drugs to mitigate this toxicity.

In a recent study, researchers identified two cathelicidins called BMAP-27 and 28 from *bovine*, they had the molecular masses of about 3.5 kDa. Both BMAP-27 and BMAP-28 had significant antibacterial properties against *P. aeruginosa* and *E. coli*, with MIC values of 1 μM and 0.25–2 μM (Skerlavaj et al., 1996). Another study found that these two AMPs have MIC_90_ values 16 and 32 μg/mL for 25 other strains of *P. aeruginosa* (Pompilio et al., 2012). Besides, BMAP-27 and 28 were not only effective in reducing the formation of *P. aeruginosa* biofilms, but also showed efficacy against biofilms that had already formed, with the concentration of 8–16 and 80–160 μg/mL, respectively (Pompilio et al., 2012). The antibacterial mechanism was related to the rapid induction of membrane permeabilization (Xie et al., 2020). However, these two cathelicidins have certain cytotoxicity to human red blood cells and neutrophils. One way to address this issue is by shortening the C-terminal of the cathelicidins, which greatly reduces their cytotoxicity without compromising their antibacterial activity.

*Human* beta-defensin 2 (HBD2) was one of the *human* β-defensins with a molecular mass of 4–5 kDa, it has been found to have multiple physiological functions (Yang et al., 1999). HBD2 showed significant antimicrobial activity against *E. coli* and *P. aeruginosa* with LD_90_ of 10 μg/mL (Schröder and Harder, 1999). At the concentration of 0.25–0.5 μM, HBD2 exhibited inhibitory effect against *P. aeruginosa* biofilm formation, although it did not inhibit metabolic activity (Parducho et al., 2020). The results also indicated that HBD2 could induce the change of the biofilm surface topology, then interfering with the transport of biofilm precursors into the extracellular space. Furthermore, HBD2 has been found to be biocompatible and safe, with no toxicity observed in hMSCs, osteoblasts, keratinocytes or HeLa cells at any concentration tested (Warnke et al., 2013).

### 2.4. AMPs from insects

Many AMPs were isolated from the plasma and leukocyte extracts of *insects*, and these have been shown to possess strong antibacterial effects (Van Moll et al., 2022). In recent years, many studies investigating the anti-*P. aeruginosa* activity of insect AMPs had been carried out, and we summarized some of the findings below.

*Royal jelly* (RJ) is a substance secreted by the hypopharyngeal and mandibular glands of *worker bees*, and it is enriched with protein, carbohydrates, vitamins, and minerals (Fujiwara et al., 1990; Fontana et al., 2004). A study has discovered novel AMPs (Jelleines) in RJ that have broad-spectrum antimicrobial activities, including gram-positive and gram-negative bacteria (Fontana et al., 2004). The molecular mass of Jelleines I-III is approximately 1 kDa. They displayed good *in vitro* antibacterial activity against *E. coli* and *P. aeruginosa*, with MIC values of 2.5–15 and 10–30 μg/mL, respectively (Fontana et al., 2004). Further investigations indicate that Jelleine-I could form aggregates that accumulated in the head group region of the membrane, leading to cell membrane disruption and leakage (Cabrera et al., 2014). Jelleine-I has been reported to have a hemolytic effect of 5–11.3% on rat erythrocytes and mouse erythrocytes, which possesses the potential for further study.

Two cecropins (Hill-Cec1 and Hill-Cec10) that were discovered in *black soldier fly* and are 4.79 and 5.28 kDa in size, had been found to effectively inhibit the growth of *P. aeruginosa* and *E. coli* planktonic cells, with the MIC values of 0.25 and 1 μM, respectively (Van Moll et al., 2022). The biofilm mass could be reduced by 50% when the concentration of Hill-Cec1 and Hill-Cec10 were 1.3 ± 0.57 μM and 7.5 ± 3.5 μM, respectively. However, they had no scavenging effect on the pre-formed biofilm. Furthermore, they exerted their antimicrobial effects by disrupting the cell membrane of *P. aeruginosa* and causing its depolarization. In addition, both cecropins showed a low hemolysis rate of less than 10% at a concentration of 64 μM, indicating a higher level of safety.

### 2.5. AMPs from amphibians

Previous studies had identified a variety of AMPs from *amphibian skin* and their antimicrobial activities had been demonstrated (Simmaco et al., 1994; Ponti et al., 1999; Luca et al., 2013). Here, we summarized the anti-*P. aeruginosa* activity of several AMPs.

Dermaseptins are a family of linear polycationic peptide consisting of 28 to 34 amino acids, which were originally isolated from the skin of *Phyllomedusa sauvagei*, a tree-dwelling *South American frog* (Mor et al., 1991). Numerous studies have confirmed that dermaseptins exhibited significant and extensive antimicrobial activities against various microorganisms, including gram-positive and gram-negative bacteria (Marynka et al., 2007; Zairi et al., 2007; Zaïri et al., 2013), fungi (Morton et al., 2007), viruses (Bergaoui et al., 2013), and protozoa (Dagan et al., 2002; Efron et al., 2002). A study demonstrated that dermaseptin K_4_S_4_ and K_4_K_20_S_4_, which have a molecular mass of about 2.8 kDa, displayed antibacterial effects against *P. aeruginosa* PA01 and *E. coli* MG1655, with MIC of 0.39–12.5 μg/mL and 0.19–0.39 μg/mL, respectively (Zaïri et al., 2014). In addition, when the concentration of dermaseptin derivatives were 2-fold of MIC, the pre-formed biofilm of *P. aeruginosa* wasn’t dissolved or disrupted, but the survival rate of biofilm cells was significantly reduced. The mechanisms of anti-*P. aeruginosa* biofilm may be similar to another dermaseptin derivative, S4(1–16) M4Ka, which inhibited biofilm formation by breaking down membrane lipids and dispersing bacteria (Quilès et al., 1858). Moreover, their CC_50_ (50% cytotoxic concentration) value was around 28 μg/mL. Although their toxicity is much lower than that of dermaseptin S4, further structural optimization is still urgent for their future study.

Wang G et al. had identified five AMPs (Temporin-LK1, gaegurin-LK1, gaegurin-LK2, rugosin-LK1, and rugosin-LK2) with molecular masses ranging from 1.95 to 3.52 kDa from skin secretions of *Limnonectes kuhlii frogs* (Wang et al., 2013). Experimental results demonstrated that they all possessed very effective antibacterial activities against *P. aeruginosa* and *E. coli* ML-35P strains, with MIC of 2.5–10 μg/mL and 10–50 μg/mL, respectively, indicating the potential to treat bacterial infections. These five AMPs also have slight hemolytic activities, causing the lysis of rabbit red blood cells in varying percentages. Temporin-LK1, gaegurin-LK1, gaegurin-LK2, rugosin-LK1, and rugosin-LK2 can induce the hemolysis of 10.2, 5.6, 6.2, 3.8, and 6.1%, respectively.

Esculentin (1–21), which was isolated from *amphibian skin* and had a size of 2.61 kDa, demonstrated efficacy on planktonic cells of gram-negative bacterial pathogen *P. aeruginosa* and *E. coli*, with MIC of 3.2 μM and 0.65 μM (Islas-Rodrìguez et al., 2009). Another study found that Esc (1–21) also exhibited antibacterial effects against MDR clinical isolates of *P. aeruginosa*, with the MICs of 4–8 μM (Luca et al., 2013). Esc (1–21) also had potent anti-*P. aeruginosa* biofilm effects, the minimum biofilm eradication concentration (MBEC) and minimum bactericidal concentration (MBCb) were 6 μM and 12 μM, respectively. Furthermore, in *in vivo* experiments, it was observed that Esc (1–21) significantly increased the survival rate of mice with *P. aeruginosa* sepsis or pulmonary infection (Luca et al., 2013). The mechanism of action showed that it can anti-*P. aeruginosa* PAO1 biofilm by penetrating biofilm plasma membrane and causing the release of β-galactosidase (Luca et al., 2013). Esc (1–21) did not show any toxicity to human erythrocytes, lung epithelial cells or mouse macrophages *in vitro* at the concentration of MIC.

Lin et al. discovered three AMPs (brevin-1HL, temporin-HLa, and temperin-HLb) from the skin secretion of *Hylarana latouchii*, and they had a molecular weight of 1.5–2.5 kDa (Lin et al., 2021). Brevin-1HL exhibited a moderate effect against *P. aeruginosa* planktonic cells and biofilm, with MIC and MBIC of 256 and 512 μg/mL, respectively. Temporin-HLa and temperin-HLb only showed a weak inhibitory effect on *P. aeruginosa*, with MIC over 512 μg/mL. The study also revealed that these three peptides induced bacterial death by destroying cell membranes and binding to bacterial DNA. Among them, only Brevinin-1HL possesses the bactericidal effects against *E. coli*, with an MBC of 128 μg/mL. However, it should be noted that Brevinin-1HL exhibits strong hemolytic activity on horse red blood cells, causing 86.8% of hemolysis at 256 μg/mL (Lin et al., 2021).

### 2.6. AMPs from other groups

In addition to the AMPs mentioned above, we have also compiled a list of AMPs with great antimicrobial activities identified from other groups in Table 1, such as *fishes*, *alligators*, *silkworm*, and *snails*. One of these AMPs, called Gaduscidin-1 (Gad-1), was originated from *Atlantic cod*, and has been shown to have antibacterial effect against *P. aeruginosa* (Portelinha and Angeles-Boza, 2021). Gad-1 exhibited inhibitory effect against *P. aeruginosa* planktonic cells, with the MIC of 2 μg/mL and 16 μg/mL at PH 6.4 and 7.4, respectively. Furthermore, Gad-1 was also effective in removing 60–72% of the pre-formed biofilm at a concentration of 64 μM. It also could completely inhibit biofilm formation when the concentration exceeding 32 μM (Portelinha and Angeles-Boza, 2021). Moreover, biofilms contain extracellular DNA (eDNA), which not only helps in stabilizing the structure of biofilms but also promotes horizontal gene transfer, ultimately leading to increased biofilm resistance to antibiotics (Whitchurch et al., 2002; Wilton et al., 2016; Nolan et al., 2020). In addition to reducing the viability of *P. aeruginosa* in biofilms, Gad-1 can cleave eDNA, making it be a possible compound for the treatment of bacterial biofilms.

Piscidins were isolated from *fishes* and could kill a variety of microorganisms, including methicillin-resistant *Staphylococcus aureus* (Salger et al., 2016). Libardo et al. had tested the antimicrobial activities of piscidin 1 and piscidin 3 with the molecular mass of approximately 3 kDa, which showed excellent activity against *E. coli* and moderate anti-*P. aeruginosa* activities, with MICs of 2–8 μM and 16–32 μM, respectively (Salger et al., 2016). It is interesting to note that although these two AMPs are similar, they possess distinct antibacterial mechanisms of action. Piscidin 1 disrupts cell membrane, while piscidin-3 forms covalent bonds with Cu^2+^ through its N-terminal amino acid and exerts nuclease activity, leading to the disruption of *P. aeruginosa* eDNA and displaying anti-biofilm activity (Libardo et al., 2017). However, both piscidins have high hemolytic activity at a concentration of 25 μg/mL.

*Crocodilian animals*, which are a group of ancient creatures, have also been studied for their antimicrobial properties (Merchant et al., 2006; Darville et al., 2010). Researchers had discovered three AMPs derived from *Alligator mississippiensis* that have varying degrees of anti-*P. aeruginosa* activity and different mechanisms of action. Apo5 and Apo6, which have molecular masses of 3.13 and 2.79 kDa, respectively, displayed moderate anti-*E. coli* effects and stronger anti-*P. aeruginosa* activities, with EC_50_ of 3.85–19.7 and 0.0878–1.17 μg/mL. The underlying mechanism may be related to disrupt membrane (Barksdale et al., 2016). While A1P, with a molecular weight of 4.13 kDa, showed weaker inhibition against *P. aeruginosa* PAO1 with an EC_50_ of 38.6 μg/mL, and stronger inhibition against *E. coli* strains with an EC_50_ of 2.51–9.2 μg/mL. The main mechanism of action was not related to the disruption of cell membranes or DNA binding (Barksdale et al., 2016). These three AMPs have high safety profiles, with minimal hemolytic effects on erythrocytes at 300 μg/mL, and no significant cytotoxic to A549 cells at the concentration of 100 μg/mL. Bishop et al. discovered eight AMPs from *Alligator mississippiensis* plasma, and had evaluated their antimicrobial effects (Bishop et al., 2015). The data showed that five AMPs (APOC1_64-88_, APOC1_67-88_, FGG_398-413_, FGG_401-413_, A1P_394–428_), which have molecular masses ranging from 1.56 to 4.11 kDa, possessed antibacterial activities against *E. coli* and *P. aeruginosa in vitro*, with EC_50_ of 0.099–0.332 μM and 0.948–11.1 μM, respectively.

ABP-CM4, a peptide with a size of 3.79 kDa, was isolated from the hemolymph of the *silkworm*, *Bombyx mori* (Li et al., 2020). It showed promising activity against several microorganisms, in particular *P. aeruginosa* ATCC 27853 and *E. coli* K_12_D_31_, with MIC of 16 μM and 12 μM *in vitro*. The main mechanism of action of ABP-CM4 was the disruption of cell membrane and interaction with DNA. Importantly, ABP-CM4 was not cytotoxic to HEK-293 cells even at a concentration of 80 μM.

The mucus secreted by *snails* also exhibits antibacterial properties. For instance, Pitt et al. demonstrated that antimicrobial substance with 30–100 kDa in size in *Helix aspersa* mucus displayed inhibitory effects against two *P. aeruginosa* strains collected by laboratory. The antimicrobial disc diffusion assay showed obvious measurable zones of inhibition, with values of 11.12 mm and 11.63 mm (Pitt et al., 2015). Furthermore, researchers discovered that AMPs with molecular masses of 17.5–37.4 kDa, identified in *Cornu aspersum mucus*, could inhibited *P. aeruginosa* strains obtained from patients with cystic fibrosis. The mean zones of inhibition recorded were all between 9 and 13 mm (Pitt et al., 2019).

After summarizing the above antibacterial effects of AMPs, we found many AMPs from different sources exhibit activity against both *P. aeruginosa* and anti-*E. coli*. In general, higher concentrations of AMPs are required to kill *P. aeruginosa* compared to *E. coli*. The MIC values of AMPs were typically smaller than those of conventional antibiotics, indicating that the antibacterial potency of AMPs was generally stronger than conventional antibiotics. Notably, PG-1, FLG2-4, Jelleines and five AMPs derived from *Limnonectes kuhlii* skin secretions all possessed significant anti-planktonic cells effects *in vitro*, with MIC values ranged from 0.3 to 10 μg/mL. Furthermore, AMPs have a distinct antibacterial mechanism compared to traditional antibiotics, which makes it difficult for pathogenic bacteria to develop resistance to AMPs. The mechanism of action of AMPs involving disrupting cell membrane structure, as seen in protegrin-1, BMAP-27 and 28, Jelleines, Hill-Cec 1, and Dermaseptin K_4_S_4_. In addition, some AMPs exerted antibacterial effects by interacting with DNA of *P. aeruginosa* and impeding the substance metabolism, such as MtDef5, FLG2-4, Brevinin-1HL, Temporin-Hla, and ABP-CM4. Additionally, the hemolytic activity of these natural AMPs is quite low, except for LL-37 and piscidins. Thus, these results of these studies have shown that AMPs had the potential to be used as antibacterial candidate. However, further research is needed to determine their effectiveness in biofilm prevention, as well as their applicability *in vivo* and in clinical settings.

## 3. Interactions of AMPs with antibiotics

The above studies showed that AMPs primarily worked by damaging bacterial membrane. This was achieved by forming pores, which disrupted the osmotic pressure balance and finally leaded to cell lysis (Zhang et al., 2001; Hollmann et al., 2018; Luo and Song, 2021). The antibacterial mechanism of conventional antibiotics involves inhibiting of DNA replication, DNA transcription, cell wall synthesis, or targeting of topoisomerases and penicillin-binding proteins (PBPs) (Abushaheen et al., 2020). Unfortunately, MDR bacteria strains can prevent the entry of conventional antibiotics into bacterial cells, resulting in antimicrobial treatment failure (Abushaheen et al., 2020).

In theory, AMPs can increase the permeability of the cytoplasmic membrane, allowing antibiotics to enter the bacterial body and exert their antibacterial effects. Some studies have demonstrated that certain AMPs, when combined with conventional antibiotics, have a synergistic inhibitory effect against human pathogens. One example is the use of colistin in combination with azithromycin, erythromycin, and clarithromycin to treat MDR *Klebsiella pneumoniae*, *P. aeruginosa* and *Acinetobacter baumannii*. This combination increased membrane permeation and helped antibiotics enter the cells, thereby inhibiting the synthesis of ribosomal proteins (Ramchuran et al., 2018). Similarly, the combination of HBD3 and LL-37 with tigecycline, moxifloxacin, piperacillin / tazobactam and meropenem has been found to have synergistic effects against *Clostridium difficile* infections (Di Luca et al., 2014). However, not every combination of AMPs and conventional antibiotics have a synergistic antibacterial effect. Some combinations may exhibit indifference or even antagonistic effects. For instance, MDL-3 derived from *housefly larvae* demonstrated an antagonistic effect against *Salmonella Typhimurium* 50,013 when combined with penicillin or streptomycin. Consequently, more research is needed to understand the mechanisms of action for each AMP and antibiotic are different, as well as the complex relationship between the drug combination and bacteria.

One of the promising approaches for treating *P. aeruginosa* resistant infections is the combination therapy with two antibacterial drugs that have a synergistic effect (Giacometti et al., 1999; Mataraci Kara et al., 2020). Previous studies have shown that a considerable number of AMPs not only show anti-*P. aeruginosa* activities alone, but also display synergistic or additive effects in combination with drugs or compounds (Giacometti et al., 1999; Cirioni et al., 2006; Hollmann et al., 2018; Lei et al., 2019; Mataraci Kara et al., 2020). We have summarized these interactions of AMP-antibiotics combinations against *P. aeruginosa* in Table 2.

### 3.1. Microorganism-derived AMPs

Jahangiri et al. had evaluated the effects of nisin (with 3.35 kDa in size) in combination with colistin against six *P. aeruginosa* strains. They found that the MIC of nisin was reduced from 128 to 256 μg/mL to 16–32 μg/mL, the MIC of colistin was reduced from 0.5–8 μg/mL to 0.125–4 μg/mL, and the FICI was 0.375–0.625, displaying a synergistic antibacterial effect against four *P. aeruginosa* strains (Jahangiri et al., 2021). In a previous study, it was observed that nisin Z combined with antibiotics had synergistic effects on *P. fluorescens* isolates (*n* = 5). No obvious antibacterial effect was observed when nisin Z used alone, while the MIC was decreased to 0.125–25 μg/mL when combined with antibiotics. Also, the MIC of antibiotics was reduced to 0.015–125 μg/mL, and FICI values were 0.01–0.5 (Naghmouchi et al., 2012).

### 3.2. Human-derived AMPs

P10, a molecule with a size of 3.11 kDa, displayed antimicrobial activity against both *Acinetobacter baumannii* and *P. aeruginosa* strains with MICs of 8–32 and 8–16 μg/mL, respectively. When P10 was combined with antibiotics (ceftazidime and doripenem), it was found to have synergistic (*n* = 4–5) or additive (*n* = 1–2) activities against *P. aeruginosa* isolates. The MIC of P10 was reduced from 8 to 16 μg/mL to 2–8 μg/mL, the MIC of ceftazidime was reduced from 4 to 64 μg/mL to 1–32 μg/mL, the MIC of doripenem was reduced from 2 to 16 μg/mL to 0.5–4 μg/mL, respectively (Jahangiri et al., 2021). LL-37 also displayed synergistic or additive bactericidal effects on *P. aeruginosa* planktonic cells when used in combination with colistin, vancomycin, and polymyxin B, with FICI of ≥0.38 (Han et al., 2022). The research further explained that the LL-37-antibiotic combination worked synergistically by increasing permeability of bacterial cell membranes, allowing antibiotics to enter *P. aeruginosa* more easily and exerted the antibacterial effects. Another study demonstrated that LL-37-antibiotics combination also displayed synergism or addition against *P. aeruginosa* biofilms. The MBEC values for antibiotics and LL-37 were 80–5,120 and ≥ 640 mg/L, respectively. When combined with LL-37 at the concentration of 64 mg/L, the MBEC value of antibiotics could be significantly reduced by 8 times (Dosler and Karaaslan, 2014).

### 3.3. Frog and crab-derived AMPs

Magainins are a kind of AMPs that originate from the skin of *African clawed frog Xenopus laevis* (Zasloff, 1987; Chopra, 1993). Numerous studies have demonstrated that magainin II has inhibitory activities on gram-negative and gram-positive bacteria, fungi, and protozoa (Jacob and Zasloff, 1994; Zairi et al., 2009). Magainin II has a molecular mass of 2.48 kDa and exhibits antimicrobial activity when used alone. Results of microbroth dilution assay showed that the MIC of magainin II against *E. coli* D31 and *P. aeruginosa* was 5 and 4 μg/mL, while rifampicin had no significant antibacterial effect when used alone. Furthermore, Cirioni and his colleagues demonstrated that magainin II in combination with rifampicin exhibited synergistic effects against *P. aeruginosa* strains both *in vitro* and *in vivo*, with a FICI of 0.312 (Cirioni et al., 2008). It is worth mentioning that *in vivo* experiments showed that the combination intervention resulted in good outcomes, including reduced mortality rates, cytokines levels, and improved treatment of bacteremia.

Lys-[Trp6]hy-a1 (lys-al) was an AMP isolated from the skin secretion of the *frog Hypsiboas albopunctatus*, with a molecular weight of 1.87 kDa (Castro et al., 2009; da Silva et al., 2013). Lys-al had the ability to inhibit *P. aeruginosa* ATCC 9027 planktonic strains, with the MIC and MBC of 125 μg/mL (Carneiro et al., 2020). Moreover, when Lys-al was used in combination with ciprofloxacin, it showed synergistic effects against *P. aeruginosa* isolates with a FICI of 0.37, the MIC of ciprofloxacin and lys-a1 was reduced by 4–8 times. Besides, the combination of ciprofloxacin/lys-a1 also displayed an additive inhibitory effect on pre-formed biofilm of *P. aeruginosa*. Furthermore, it was observed that the shape of the cells had changed and the surface had become uneven after treatment with ciprofloxacin/lys-a1. Researchers speculated that cell membrane damage could lead to cytoplasm leakage or increase the possibility of antibiotic entry, ultimately resulting in synergistic antimicrobial effects.

Bessa et al. demonstrated that ocellatinPT3 (with a molecular weight of 2.53 kDa), an AMP isolated from the skin secretion of the *frog Leptodactylus pustulatus*, possessed inhibitory activities against planktonic cells and biofilms of *P. aeruginosa* (Oliveira et al., 2016; Bessa et al., 2018). When ocellain-PT3 combined with ciprofloxacin and ceftazidime, it showed synergistic effects on MDR strains, the MIC of them could be reduced 4–8 times, and the FICI was between 0.25 and 0.5. Furthermore, ocellain PT3 with a concentration over 256 μg/mL could inhibit the biofilm formation of *P. aeruginosa*. Ocellatin-PT3 exerted its antimicrobial effects primarily by acting on the LPS of *P. aeruginosa*.

Colistin is the last line of AMP antibiotic in clinical practice, and it is generally used in combination with other antibiotics to treat MDR bacterial infection (Petrosillo et al., 2008; Park et al., 2016; Almutairi, 2022; Xie et al., 2022). AMPs called Citropin 1.1 and temporin A with molecular weights of 1.62 and 1.4 kDa were secreted by the dorsal gland and submental gland of *Litoria citropa* and the skin of *European red frog Rana temporaria*, respectively (Simonetti et al., 2008; Ghiselli et al., 2011). Previous research by Jorge et al. demonstrated that colistin-citropin 1.1 combination and colistin-temporin A combination both had synergistic and additive inhibitory effects against *P. aeruginosa* planktonic cells, with the FICI range from 0.26 to 0.75 (Jorge et al., 2017). Similar synergistic and additive effects were also observed against *P. aeruginosa* biofilms. In addition, Pamp SJ et al. found that colistin was effective in killing cells with low metabolic activity in *P. aeruginosa* biofilms (Pamp et al., 2008). Ciprofloxacin and tetracycline could kill metabolically active biofilm cells, therefore, when colistin combined with these two antibiotics, a significant synergistic effect was observed, resulting in the complete eradication of almost all biofilm cells of *P. aeruginosa* (Pamp et al., 2008). NCL195 is a new antibiotic with little cytotoxicity, which belongs to the analog of robenidine (Ogunniyi et al., 2017). It exerted obvious antibacterial activity against *Streptococcus pneumoniae*, *Staphylococcus aureus*, *A. baumannii*, and *K. pneumoniae* strains by disturbing their cell membrane potential, with the MIC ranged from 0.25 to 8 μg/mL (Ogunniyi et al., 2017). Recently, Nguyen and his colleagues found that the combination of NCL195 and colistin exhibited significant synergism against *P. aeruginosa* (n = 18) planktonic cells with a time- and concentration-dependent manner, the MIC of NCL95 was reduced from >256 to 0.5–4 μg/mL, the MIC of colistin was reduced from 0.25–2 to 0.03–1 μg/mL, and the FICI index was 0.12–0.5 (Nguyen et al., 2021). In addition, the researchers also observed that the combination of NCL195-colistin caused more cell membrane damage to the cell membrane compared to colistin alone, which was considered as the synergistic antibacterial mechanism.

Tachyplesin I was originated from the blood cells of *Tachypleus tridentatus*, it had a molecular weight of 2.27 kDa and possessed extensive bactericidal abilities (Xie et al., 2016). It is found that tachyplesin I in combination with colistin had the synergistic and additive inhibitory activities against *P. aeruginosa* planktonic cells, the MICs of them were reduced by 4–8 times and 2–8 times, respectively. Similarly, they also exhibited synergism and addition against biofilms (Jorge et al., 2017). Another AMP called sphistin, with the molecular mass of 3.96 kDa, was derived from the *mud crab Scylla paramamosain*. It showed potent synergistic activity when used in combination with azithromycin and rifampicin against *P. aeruginosa* planktonic cells, the MICs of antibiotics were reduced from 180 to 18 and 2.5 to 0.625 μg/mL, respectively (Chen et al., 2015; Liu et al., 2020). The MIC of AMP was decreased from 24 to 1.5–6 μmol/L, and the FICI was below 0.35 (Liu et al., 2020). When sphistin combined with the two antibiotics, the cell membrane permeability of *P. aeruginosa* was increased, which facilitated the uptake of antibiotics and allowed them to display antibacterial effects.

### 3.4. Others

AMP melittin (with 2.87 kDa in size) is the main constituent of *bee* (*Apis mellifera*) venom and has broad-spectrum antibacterial activities (Li et al., 2017). The MIC values of melittin against *P. aeruginosa* were ranged from 1 to 8 μg/mL, while the MIC values against *Acinetobacter baumannii* were even lower at 0.25–0.5 μg/mL. Many reports demonstrated that it also possessed synergistic effect in combination with some conventional antibiotics (such as vancomycin, oxacillin, and amikacin) against MDR strains (Al-Ani et al., 2015). Akbari et al. reported that melittin exerted synergism against MDR *P. aeruginosa* strains when combined with doripenem and ceftazidime with the FICI of 0.01–0.5 (Akbari et al., 2019). The MIC of antibiotics was reduced from 8 to 64 μg/mL to 0.12–1 μg/mL and 4–64 μg/mL to 0.5–8 μg/mL, the MIC of AMP was reduced from 1 to 8 μg/mL to 0.03–0.5 μg/mL, as well as cytotoxicity was reduced by 100-fold.

LfcinB is a 25-amino acid peptide derived from *bovine lactoferricin*, which possessed a broad spectrum of phycological activity (Román et al., 2019). The AMP LfcinB (20–25)_4_ was a short peptide with 4.84 kDa in size derived from LfcinB, which has been demonstrated to have antibacterial activity against *P. aeruginosa* and *E. coli*, with MICs of 11 μM and 5–22 μM, respectively. When the concentration of LfcinB (20–25)_4_ was 11 μM, it exhibited a 14% hemolytic activity. When LfcinB (20–25)_4_ combined with ciprofloxacin, it showed synergistic effects against *P. aeruginosa* (Vargas-Casanova et al., 2019). The MIC of AMP was decreased from 100 to 3.1 μg/mL, the MICs of ciprofloxacin were reduced from 0.4 to 0.02 μg/mL, and FICI was 0.09.

OH-CATH30, a peptide isolated from the *king cobra* with a size of 3.61 kDa, exhibited antibacterial activity with MIC values of 3.125–25 μg/mL against *P. aeruginosa* and MIC values of 1.56–12.5 ug/mL against *E. coli* (Zhao et al., 2008; Li et al., 2012). OH-CATH30 had a low hemolytic activity against human red blood cells, even at a high concentration of 400 μg/mL. It also exhibited low cytotoxicity against HaCaT cell lines, with an LD_50_ (half-lethal dose) above 200 μg/mL (Li et al., 2012). Moreover, studies of the interaction between the peptide and ciprofloxacin/levofloxacin demonstrated synergistic effects against four *P. aeruginosa* isolates, with the FICI value of 0.375–0.5 (Li et al., 2014).

Cecropin A2 is a 36-residue α-helical cationic AMP with 3.6 kDa in size derived from *mosquito Aedes aegypti*, and it possessed antibacterial activities (Zheng et al., 2017). The AMP exhibited little hemolytic activity and toxicity toward mammalian cells. The MICs against clinical *P. aeruginosa* isolates were found to be 32–64 μg/mL, while the MICs against other gram-negative bacteria ranged from 2 to 32 μg/mL. The combination of cecropin A2 and tetracycline exerted synergistic activities against the planktonic cells of eighteen *P. aeruginosa* strains, and the MIC of AMP and antibiotic was reduced by 8-fold, with a FICI value of 0.25. Furthermore, the intervention of Cecropin A2-tetracycline combination also showed synergistic protection in *G. mellonella* models *in vivo* experiments. The researchers found that Cecropin A2 could bind to the LPS of *P. aeruginosa*, causing membrane permeation and interacting with bacterial DNA, thereby facilitating the transfer of tetracycline to the cytoplasm. In addition, an earlier study proved that the combination of cecropin A (with the molecular mass of 3.88 kDa) and rifampicin also had synergistic inhibitory activity against *P. aeruginosa* strains, with the FICI index of 0.312 < 0.5 (Cirioni et al., 2008).

A study found that CAMA, a cecropin (1–7)-melittin A (2–9) *amide* with the molecular weight of 1.77 kDa, exerted a strong synergism when used in combination with ciprofloxacin against preformed biofilms of *P. aeruginosa* (*n* = 4) (Dosler and Karaaslan, 2014). After treatment with this combination, MBEC values of antibiotic and CAMA were reduced 8 times and 10 times, respectively.

The existing AMPs can be designed in a more thoughtful way to enhance chemical and physical properties, leading to improved effectiveness. Studies have shown that certain *synthetic* or *semi-synthetic* AMPs exhibited more significant synergistic activities against *P. aeruginosa* planktonic cells and biofilms when used in combination with traditional antibiotics (Martinez et al., 1861; Rudilla et al., 2016; Maisetta et al., 2017; Al Tall et al., 2019; Yasir et al., 2020). For example, the combination of AMP 38 (a novel *synthetic* cyclodipeptide analog of polymyxin) with imipenem had synergistic activities against imipenem-resistant *P. aeruginosa in vitro*, with a FICI of 0.07–0.18 (Rudilla et al., 2016). The AMP-antibiotic combination also exhibited a synergistic effect against biofilms, the MBEC of them were reduced from ≥500 to 62.5 μg/mL, and the FICI was 0.25. Cytotoxicity tests showed that mice administered 100 and 200 mg/kg AMP 38 survived without signs of toxicity, and it had an LD_50_ value of 283 mg/kg. The synergistic antibacterial mechanism was similar to the above-mentioned mechanisms, AMP 38 destroyed cell membrane, and facilitated the entry of imipenem, which subsequently exerted its antibacterial effect at low concentrations. The synthesis of AMPs provides new insights for fighting against MDR strains, since an unlimited number of new compounds with antibacterial effects can be designed.

Based on the above studies, we found that most of the AMPs showed significant synergistic anti-*P. aeruginosa* effects *in vitro* when used in combination with antibacterial drugs. The combination therapy reduced the effective concentration of antibiotics, expanded the range of action of antibiotics, and improved the efficacy of antibiotics, suggesting the feasibility of drug combination strategy. Studies have shown that the synergistic antimicrobial mechanisms of AMP in combination with conventional antibiotics mainly include the following four mechanisms: (1) AMP could inhibit membrane ion channels, prevented the pumping out of antibiotics, or inhibited antibiotic-degrading enzymes’ activities (Dey et al., 2021); (2) AMP could load antibiotics and promote the uptake of antibiotics by bacteria to broaden the antibiotic’s antimicrobial spectrum; (3) AMP could interact with microbial membranes to increase the permeability, which in turn allowed more antibiotics access to the interior of the bacteria; (4) AMP could enhance the anti-biofilm activity of antibiotics by interfering with signaling pathways involved in biofilm formation and maintenance (de la Fuente-Núñez et al., 2014, 2015). In this article, we have summarized the synergistic anti-*P. aeruginosa* mechanisms of LL-37, lys-al, Ocellatin-PT3, NCL195, sphistin, Cecropin A2 and AMP 38, they could increase the cell membrane permeability by disrupting the cell membrane or binding to LPS, which increased the opportunity of antibiotics into bacterial interior. However, most of the current studies on AMPs-antibiotic synergistic antimicrobials did not have enough sufficient clinical data, therefore, in-depth studies on AMPs combined with antimicrobials against *P. aeruginosa* need to be carried out in the future.

## 4. Clinical studies on some AMPs

With the increase in research, some AMPs had entered clinical trials, while most required more detailed and validated studies to learn about their antimicrobial activity and mechanism of action. To date, at least 20 AMPs had entered clinical trials (Mercer and O’Neil, 2013).

For example, Pexiganan, which possessed potent antibacterial activities *in vitro*, has passed two phase III clinical trials, demonstrated its efficacy, safety, and effectiveness against infected diabetic foot ulcers (Lipsky et al., 2008). In addition, Omiganan exhibited rapid and potent antibacterial activity by disrupting the bacterial plasma membrane. Currently, a 1% gel product of Omiganan (Omigard) was in a phase III trial to demonstrate its efficacy in the prevention of catheter-related infections. Besides, Omiganan had also been used in the treatment of rosacea and has successfully completed a phase II trial (Fritsche et al., 2008; Kang et al., 2014). Brilacidin had potent bactericidal activity against both resistant gram-positive and gram-negative bacteria. In 2014, a phase II trial enrolled 215 patients with acute bacterial skin and skin structure infections caused by MRSA and found that the therapeutic efficacy of Brilacidin administered intravenously was comparable to daptomycin (Méndez-Samperio, 2014). POL7080, a mimic of the antimicrobial peptide Protegrin-1, had anti-MDR *P. aeruginosa* effects both *in vitro* and *in vivo*. Currently, POL7080 had successfully completed Phase I clinical trial for the treatment of *P. aeruginosa* infections, demonstrating the safety and good tolerability (Tillotson and Theriault, 2013; Romano et al., 2019). There are also many AMPs that have passed Phase I/II clinical trials, but failed to obtain satisfactory results in Phase III trials, such as Iseganan (Giles et al., 2004; Trotti et al., 2004; Kollef et al., 2006; Elad et al., 2012; Mercer and O’Neil, 2013). Moreover, several AMPs, such as Plectasin NZ2114 and MU1140, had exhibited remarkable therapeutic efficacy in preclinical researches, and they held great promise for further clinical development (Marr et al., 2006; Ghobrial et al., 2009; Breidenstein et al., 2015).

## 5. Discussion

In this review, we summarized the anti-*P. aeruginosa* activities of AMPs used alone and interactions with some conventional antibiotics. *In vitro* studies have shown that certain AMPs display obvious inhibitory effects against *P. aeruginosa* planktonic cells with MICs ≤10 μg/mL, AMPs exerted their antimicrobial effects mainly by disrupting bacterial cell membranes or interacting with DNA. Moreover, a considerable number of AMPs-antibiotics combination have significant synergistic inhibitory effects against *P. aeruginosa* planktonic cells, as well as the biofilm formation. *In vivo* studies, some combinations have also increased the survival rate and reduced the extent of infection among infected animal models. The AMPs-antibiotics synergistic combination not only helped to reduce the individual concentration and broaden the antibacterial spectrum, but also decreased drug resistance, toxicity, and other side effects. Most of the synergistic mechanisms of action were that AMPs could increase cell membrane permeability, making it easier for antibiotics to enter bacterial interior, and then exerted their antimicrobial effects. Although the synergistic antibacterial effects of AMP in combination with antibiotics had been demonstrated *in vitro*, the validation of their clinical synergistic activity has been rarely studied due to its limitations and different pharmacological properties from antibiotics. In addition, it is worth noting that some AMPs have been found to exert their antimicrobial effects through a combination of membrane-targeted and non-membrane-targeted modalities. For instance, arenicin-3 is a novel AMP that not only binds to the outer and cellular membranes of bacteria and disrupts their integrity, but also transfers to the cytoplasm to inhibit protein synthesis, ultimately exerting significant anti-gram-negative effects (Ciumac et al., 2019). Therefore, the cell membrane is no longer the only target of AMPs against pathogens. AMPs can act on the cell wall or intracellular nucleic acids, proteins, enzymes, or organelles to inhibit crucial intracellular processes, then leading to metabolic inhibition of the pathogens and causing bacterial death. Further research on the intracellular target of AMPs is of great significance in preventing the development of resistance in pathogens.

Although AMPs have potential therapeutic benefits compared with traditional antibiotics, they also have certain limitations, which hinder the clinical development and application. AMPs extracted naturally have low purity and quantity, as well as poor absorption, distribution, metabolism, excretion characteristics, low permeability and solubility (Di, 2015). A great number of researchers found that some properties of AMPs can be improved by changing peptide composition, making post-translational modification, recombining AMPs, and using computer-assisted discovery and design, which were meaningful to translate AMPs into useful clinical candidates for development (Irazazabal et al., 1858; Matsuzaki, 2009; Deo et al., 2022). Researchers usually use a variety of methods to design efficient, low-toxicity, and stable AMPs. These methods include: (1) replacement of amino acids in the peptide sequence with non-natural α-amino acid derivatives, such as D-amino acids, hexafluoroleucine, and α-amino-guanidinopropionic acid; (2) N-terminal acetylation or C-terminal amidation; (3) dimerization and disulfide bond cyclization; (4) optimization of physicochemical properties. Focusing on these studies will provide valuable insights into the application of AMPs in complex disease and lay a solid foundation for innovative drug development.

## 6. Conclusion

Natural AMPs have anti-*P. aeruginosa* and *E. coli* activity with low MIC values when used alone, and they act primarily by interacting with cell membranes or targeting organelles within the cell. Membrane-targeted antimicrobial mechanisms include four models. In this review, the antimicrobial mechanisms of Ocellatin-PT3 and Cecropin A2 are associated with LPS binding; the antimicrobial mechanisms of ACD and defensin-d2 are associated with an increase in the level of ROS. And the profound antimicrobial mechanisms of many other AMPs still need to be further discussed. In addition, based on the study of the antibacterial effect of natural AMPs alone or in combination with antibiotics against *P. aeruginosa in vitro*, it is still necessary to explore the synergistic antibacterial mechanism further or continue to develop AMPs structural analogs. Besides, it is imperative to further ensure the efficacy and safety of combined treatment *in vivo* animal models, which will be of great significance against clinical *P. aeruginosa* infection.

## Author contributions

XC collected and analyzed the data of the review, and wrote the whole review and created Tables 1, 2. SS, YY, and LY helped with it. LH contributed to the writing to this article. All authors have reviewed and approved the manuscript. All authors contributed to the article and approved the submitted version.

## Conflict of interest

The authors declare that the research was conducted in the absence of any commercial or financial relationships that could be construed as a potential conflict of interest.

## Publisher’s note

All claims expressed in this article are solely those of the authors and do not necessarily represent those of their affiliated organizations, or those of the publisher, the editors and the reviewers. Any product that may be evaluated in this article, or claim that may be made by its manufacturer, is not guaranteed or endorsed by the publisher.
